# Who is in the driving seat? Assessing innovation performance feedback and digital transformation of manufacturing enterprises

**DOI:** 10.1016/j.heliyon.2025.e42671

**Published:** 2025-02-13

**Authors:** Dongwei Li, Jaffar Abbas, Haojun Wang, Khalid Al-Sulaiti, Bei Lyu

**Affiliations:** a School of Business Administration, South China University of Technology, Guangzhou, China; b School of Political and Economic Management, Guizhou Minzu University, Guiyang, China; c School of Media and Communication, Shanghai Jiao Tong University, Shanghai, China; d Al Rayyan International University College in Partnership with University of Derby UK, Doha, Qatar; e School of Economics and Management, Huaibei Normal University, Huaibei, China; f Chinese Graduate School, Panyapiwat Institute of Management, Nonthaburi, Thailand

**Keywords:** Enterprise digital transformation, Innovation expectation gap, CEO power, CEO traits

## Abstract

The use of digital technologies has enabled enterprises to enhance their innovation capabilities and reconstruct their competitive advantage throughout the wave of digital transformation. Previous research has examined the driving forces behind digital transformation through the lens of institutional theory, resource-based views, dynamic capacities, and upper echelons theory. However, these studies have ignored the fact that the decision makers with bounded rationality formulate digital transformation strategies based on “heuristic decisions”. In order to address the above research gap, this study examines the impact of innovation expectation gap on digital transformation of enterprises based on the behavioral theory of the firm. In this paper, Chinese A-share manufacturing enterprises listed from 2010 to 2021 are used as the research sample. The results show that as the innovation expectation gap widens, enterprises will be more motivated to implement digital transformation to improve their innovation performance. Accordingly, the innovation expectation gap has a positive impact on the digital transformation of enterprises. After CEO power and CEO traits are embedded in the research framework, it is found that CEO power can strengthen the positive impact of innovation expectation gap on enterprise digital transformation. In addition, if the CEO has both functional diversity and digital literacy, CEO power's strengthening effect on the relationship between innovation expectation gap and enterprise digital transformation can also be enhanced. The study provides new insights for the study of enterprises digital transformation decision-making from the perspective of innovation expectation gap, which helps to expand the research topics of the behavioral theory of the firm.

## 1 Introduction

As a systematic and continuous process of strategic change, enterprise digital transformation relies on the integration of digital technologies into a wide range of key business functions, including strategic direction, organizational structure, production processes, and business models [1,2]. In addition to optimizing and reconfiguring traditional factors of production [3], this process also creates new combinations of factors of production through the incorporation of data, which results in new production functions. In response to this shift, two core digital ecosystems have emerged: innovation orientation and digital enablement, which contribute to rapid iteration, continuous optimization, and the development of data-driven innovation systems [[4], [5], [6]]. As China's economy shifts from a stage of rapid growth to a stage of high-qauality development, the real economy is facing more intense market competition than ever before. Digital transformation has become an important way for enterprises to explore new growth opportunities and development models. The Chinese government attaches great importance to the development of the digital economy and has put forward a series of strategic initiatives, such as the 14th National Five-Year Plan, which explicitly proposes to create a new advantage in the digital economy [7].In order for manufacturing enterprises to achieve high-quality development, the digital transformation and upgrading are unavoidable decisions prompted by objective circumstances [8]. Nevertheless, there exist significant disparities in the cognitive comprehension and adoption of digital transformation across various enterprises [9]. Prior researchers have consistently highlighted the influence of external institutional pressures and shifts in market demand on the digital transformation of enterprises. This has been examined from the perspectives of industrial policy, industry competition, digital technology iteration, and consumer market demand [[10], [11], [12]]. Other researchers have examined the factors that contribute to enterprise digital transformation at the micro-organizational and individual levels, including resources and capabilities [13,14], executive cognitive structure [15], and the role of Chief Digital Officers [16]. However, existing research ignores the fact that decision makers in limited rationality make “heuristic decisions” based on the “satisfaction principle”. Especially in the early stages of the digital transformation, decision makers lack a comprehensive understanding of the digital business environment and information. In addition, smart manufacturing has increased the complexity of the manufacturing system and the uncertainty of the production process, which further impacts the rational thinking of the enterprise's digital strategy [8].

Greve [17] states that the behavioral theory of the firm provides a unique theoretical framework for understanding how manufacturing enterprises make their decisions regarding digital transformation in response to risks and uncertainties. Based on this theory, when an organization fails to achieve its preset expected goals, it often triggers a mechanism known as “problematic search,” which prompts the company to adjust its established procedural decisions, thereby stimulating a strong willingness to act and a spirit of adventure in order to achieve the set goals. Furthermore, if the company's performance exceeds its goals, its subsequent decisions will tend to preserve its competitive advantage by maintaining the current stable state [17]. The behavioral theory of the firm has been extensively applied to the study of firm strategic change in the context of feedback to financial performance [18]. However, for technology-intensive manufacturing firms, the core of building sustainable competitive advantage and maintaining stable business performance lies in ensuring that innovation activities are carried out along a performance path that is efficient and aligned with the firm's goals. In such firms, innovation goals are often prioritized over financial goals, which leads them to implement innovative strategies that are forward looking and take risks when faced with innovation challenges [19,20]. Consequently, manufacturing firms cannot ignore innovation performance feedback when developing their strategic plans [[21], [22], [23]]. From this perspective, reevaluating innovation goals can help to enhance our understanding of the underlying decision-making logic of manufacturing enterprises in response to uncertainty. Unfortunately, there is a lack of literature that provides definitive explanations for the factors that promote digital transformation in manufacturing firms from the perspective of innovation performance feedback.

Amidst the complex backdrop of increasingly fierce global industrial competition, frequent disputes over core technology intellectual property rights, and the rise of trade protectionism, while many Chinese manufacturing enterprises have made significant progress, their innovative R&D capabilities have not yet been fully unleashed, and they have not yet reached the expected leading level [24]. During the critical transition period of China's economy moving towards high-quality development, the urgent need for innovative development of manufacturing enterprises has become increasingly prominent [25]. The only way to promote China's economic transformation and upgrading is to take innovation-driven development as the engine and continuously improve the efficiency of the national innovation system [25]. Therefore, decision makers often interpret a large gap between innovation expectations and actual performance as a “frustrating” evaluation of established goals and industry benchmarks for innovation. Not only does this condition affect the firm's market position, but it can also adversely affect its external image and legitimacy [26]. Research suggests that when faced with innovation expectation gaps, decision makers will enhance the diversity and novelty of technology searches, seek R&D alliance cooperations, and utilize external resources such as venture capital to achieve externalization of R&D [19,27]. Considering the importance and necessity for manufacturing enterprises to benefit from digital transformation [5], it is expected that the greater the distance between manufacturing enterprises and their anticipated level of innovation performance, the more likely they will reorganize their innovation system through digital transformation in order to improve their poor innovation performance.

As the main character in decision making in regard to innovation performance feedback [28], a CEO steers the digital transformation of an enterprise, and plays a crucial role during the consolidation of understandings and thinking models of the entire enterprise regarding digital development, expounding its strategic relevance and allocating resources [29,30]. Hence, disregarding the crucial contribution of the CEO would inevitably result in a lack of a comprehensive and precise understanding of the process involved in formulating and implementing strategic decisions for digital transformation in organizations from the perspective of innovation performance feedback. According to recent research, CEOs with greater power are more likely to give full play to their own will when making corporate strategic decisions, thereby reinforcing or hindering search decisions for performance feedback [18]. This study further explores the impact of CEO power on the relationship between the innovation expectation gap and enterprise digital transformation. It aims to gain a better understanding of the influence of CEO power in shaping digital transformation strategies. Furthermore, the upper echelons theory states that CEO traits affect their selective understanding of limited information, which in turn affects the strategic behavior of the firm [31]. Since digital transformation is a deep adaptive adjustment of assets, businesses, and organizations driven by the strategy, while the technology plays a crucial role, the initial stage of digital transformation is typically determined by the level of awareness and mindset of the organization and its executives [15]. This suggests that CEOs' own traits may also have an additional impact on how CEO power affects the relationship between innovation expectation gap and enterprise digital transformation. As a result, this study will also examine the secondary moderating effects of CEO functional diversity (which represents the CEO's ability to integrate knowledge) and CEO digital literacy (which represents the CEO's knowledge and skills in digital technology) within the theoretical framework. This analysis aims to thoroughly investigate the boundary conditions of CEO power in moderating the relationship between innovation expectation gap and enterprise digital transformation.

Based on the behavioral theory of the firm, this paper attempts to analyze the logical relationship between innovation expectation gap and digital transformation of manufacturing enterprises, using China's A-share listed manufacturing enterprise (2010–2021) as the research sample. Specifically, this paper addresses the following key questions: First, will the innovation expectation gap of manufacturing enterprises trigger enterprise digital transformation? Second, how do we leverage CEO power to influence the relationship between innovation expectation gap and enterprise digital transformation? Third, do CEO traits like functional diversity and digital literacy influence the moderating role of CEO power in the relationship between innovation expectation gap and enterprise digital transformation? By exploring the above research questions, the research contribution of this paper lies in: Firstly, it reveals the deep connection between the innovation expectation gap and enterprise digital transformation, and provides a new theoretical framework for analyzing the motivations driving the digital transformation strategies of manufacturing enterprises. Secondly, CEO power is integrated into the logical framework of innovation performance feedback. This elucidates the procedural mechanism of decision-making for enterprise digital transformation in light of innovation performance feedback, and expands the boundary conditions of the relationship between behavior drive and organizational decision-making. Finally, this paper further enhances the complexity of micro-mechanisms related to enterprise digital transformation by exploring the secondary moderating effects of CEO traits (i.e. CEO functional diversity and CEO digital literacy).

## 2 Theory and hypothesis development

### 2.1 Innovation expectation gap and digital transformation

As digital technologies develop rapidly, their generativity, malleability, and combinability are driving organizational innovation. These characteristics not only challenge existing knowledge structures, but also complement existing capabilities, playing an important role in the search and reorganization phases of the idea generation process. Specifically, digital technologies drive iterative innovation of products and business processes in various ways, such as functional embedding, transformation and upgrading, and resource integration, contributing to continuous innovation momentum within the organization [5]. Although manufacturing enterprises are actively engaging in digital transformation, they are faced with significant challenges, such as the lack of a clear blueprint and action guidelines for transformation, the disruption of the industry brought by digital technology, and the difficulty of balancing short-term performance with long-term value [8]. As a result of these factors, digital transformation entails greater risks and uncertainties [9]. According to the behavioral theory of the firm, decision makers are typically limited-rational problem solvers who often establish simple rules for making decisions [17]. When an organization's actual innovation level does not meet its expectations, its innovation development state will be considered a loss. Consequently, this triggers decision makers' risk propensities and organizational change, which results in the search for alternative solutions to restore innovation to a satisfactory level [27,32]. In this paper, we hypothesize that firms' strategic decisions regarding digital transformation are driven by the innovation expectation gap. The specific reasons are as follows:

On the one hand, the innovation expectation gap will increase the perceived pressure to acquire technological expertise. This will lead to enterprises conducting long-distance innovation searches through digital technology. Consequently, the organization will be under increasing pressure to acquire new knowledge due to its unsatisfactory innovation performance. As this pressure grows and reaches a critical threshold, it will eventually surpass short-term considerations, resulting in an expansion of the scale and scope of innovation searches [26]. Digital transformation is distinguished by its inherent self-growth, wide availability, and remarkable scalability. These characteristics enable enterprises to reshape their core strategy so that they can focus more on promoting deep interactions, cross-border collaborations, and establishing symbiotic relationships among different participants. This transformation enables enterprises to break through traditional barriers to innovation search and resource constraints [3]. Furthermore, they can utilize both internal and external innovation resources to their fullest extent, efficiently integrate external resources, and therefore develop and implement innovative products and services in accordance with market development trends [33]. Therefore, the poor innovation performance will drive a robust desire to learn about digital technology within the organization, in order to conduct innovation search and knowledge transfer across organizational units through digital transformation, aiming to overcome the current market competition and external legitimacy challenges.

On the other hand, the innovation expectation gap forces decision makers to enhance their willingness to take risks and explore more daring and imaginative digital solutions in order to address the current challenges in innovation. As the innovation expectation gap widens, it suggests that manufacturing enterprise will increasingly confront poor financial performance and possibly even the hazard of extinction in the future [19]. This will prompt key stakeholders, such as the board of directors and shareholders, to challenge and express discontent with corporate decision makers, thereby compelling decision makers to promptly seek solutions to enhance innovative performance [21]. Consequently, when decision makers are faced with the need to significantly improve performance and the possibility of being fired, they are more likely to enhance their abilities to take risks [20]and increase the intensity of problem searching to examine digital practices. When conventional solutions fail, companies may adopt riskier and more assertive coping strategies to explore innovative and potentially fruitful courses of action. This allows them to acquire new technological knowledge and bridge the gap in innovation performance [34]. Despite of twists and turns on the road to digital transformation, the innovation-driven model with digital transformation as its core has undoubtedly piloted the strategic adjustment that a manufacturing enterprise launches to acquire the external knowledge necessary for improving innovation performance and retaining competitive advantages. Given the intense competitiveness of the current business environment, if a enterprise's innovation performance falls below expectations or industry standards, its strategic decision-making will tend to be more risk-taking. This, in turn, enhances the determination and courage of enterprises to implement the digital transformation strategy, leading to a shift in its mindset and a willingness to allocate more resources towards digital initiatives. According to the aforementioned reasoning, this paper forecasts that enterprises that are further away from their desired level of innovation performance are more inclined to adopt a digital transformation strategy. This strategy involves using digital technology iteration and restructuring the innovation process to bridge the gap in innovation performance. Accordingly, it is proposed that.

H1 innovation expectation gap is positively associated with enterprise digital transformation.

### 2.2 Moderating effect of CEO power

In an enterprise, a CEO steers the generation of its strategic decisions and has the authority to convert their personal goals into practical business plans [35]. Prior research has demonstrated that performance feedback is a mechanism that generates information, and decision-makers usually adjust enterprise strategic decisions based on the difference between current performance and expected performance [18]. In this regard, the power of the decision-maker plays a crucial role in the selection and implementation of risk-taking decisions as well as their subsequent resource allocation strategies [36]. This article suggests that the power of CEOs can increase their drive to take risks and allocate resources, thereby strengthening their motivation to address the innovation gap through digital transformation strategies. First, high-power CEOs are more optimistic and tend to focus on the expected returns of digital transformation, which can bolsters the motivation of enterprises to rectify subpar innovation performance through digital transformation. Based on the approach-inhibition theory of power, individuals with high power are more likely to experience good emotions, pay attention to rewarding information, and engage in automatic information processing. This might lead to an increased tendency to seek out and take risks. On the contrary, low power activates the behavioral inhibition system. In turn, ensuing negative emotions are reflected in cognition and behaviors pertaining to punishment, threats and social restrictions [37]. Therefore, when a CEO is given higher power, their behavioral approach and risk preferences will be stimulated. As Boustanifar et al. [36] pointed out in their study, a CEO with higher power may be a risk seeker. As CEO power is augmented, the escalating risks in corporate strategic decisions would be generated. As a result, CEOs would be more prone to take high-risk decisions as a result of being enchanted by enormous benefits from risk projects. When faced with innovation dilemmas, a CEO with low power may be inhibited from taking proactive search behaviors due to such traits as loss avoidance and risk aversion [38]. Secondly, the greater the power of the CEO, the stronger the CEO's ability to influence the direction and extent of corporate strategic decisions. As the navigator of the enterprise digital transformation, a CEO is empowered to allocate enterprise resources. Consequently, we could consolidate strategies and strengthen leadership by reducing internal games and bargaining among stakeholders of an enterprise [39]. This implies that CEOs with high power are able to more accurately evaluate the level of risk involved in their strategic choices, possess greater control over resource allocation, and reduce the opposition to implementing digital transformation inside the organization. Therefore, with more power, the CEO can address the innovation expectation gap by allocating resources to implement a digital transformation strategy. Accordingly, this study proposes.

H2 The positive association between innovation expectation gap and enterprise digital transformation is stronger under CEO power.

### 2.3 Secondary moderating effect of CEO characteristics

An important question arising from the aforementioned argument is what CEO traits can further strengthen the CEO's motivation to utilize his power to implement digital transformation particularly in response to subpar innovation performance. According to previous studies, CEOs who have technical expertise are able to discern the trajectory of technological advancements within an enterprise and effectively leverage their authority to make informed decisions on technology investments, thereby mitigating the risk of pursuing technology development without a clear strategic direction [40]. This paper aims to support and expand on the previous viewpoint by examining two specific traits of a CEO-- functional diversity and digital literacy. It further analyzes the boundary conditions of CEO power based on the impact of the innovation expectation gap on processes of strategic decisions regarding enterprise digital transformation. This study speculates that when the CEO has functional diversity and digital literacy, CEO power will have a stronger promoting effect on the behavioral tendency of enterprises to respond to innovation expectation gaps through digital transformation strategies.

CEO functional diversity refers to the different professional and organizational functional experiences that a CEO has [41]. The upper echelons theory suggests that managers' career experiences will be deeply imprinted on managers’ cognitive levels, thinking habits, and behavioral patterns [31]. Since digital transformation is more than just a digital iteration of technology or equipment, it is a systematic cross-functional reform that involves all businesses of an enterprise [39], the enterprise suffering from its poor innovation performance would be more greatly motivated by its highly empowered CEO to initiate digital transformation. However, if the CEO does not have sufficient knowledge of digital technology, business processes, operating models, organizational structures, customer demands, digital management and other aspects involved in digital transformation, the CEO will be less likely to be able to integrate top-level conception of digital strategy with business processes. On the contrary, a CEO with experience in diverse functions often has a broader vision, wider knowledge scope and stronger information processing capabilities. Thus, the development of corporate decisions on risk taking and innovation would suffer impacts therefrom [42,43]. Consequently, CEOs who are complicit in digital strategic decisions are more likely to be confident in addressing risky affairs, more willing to invest in ventures, and more focused on innovation investments with long-term effects [42]. More importantly, they possess more heterogeneous knowledge resources [43] and have a clearer cognitive framework for digital projects. As a result, the CEO would be enabled to instill more confidence in expansion of innovation search, the power allocation would be secured for specific digital solutions. Furthermore, the organization would be pushed to speed up collaboration between digital technology and original business processes, so that adversity arising from poor innovation performance can soon be resolved.

As for a CEO, digital literacy refers to a set of his or her knowledge and skills in connection with digital technology. It is mainly manifested in the comprehensive abilities to collect and integrate digital resources, appropriately use digital tools and facilities, and give fully play to digital technology [30]. On the one hand, CEOs with digital literacy have information advantages and management advantages related to digitalization, which enhances their willingness to take risks in making strategic decisions connected to innovation, as well as their ambition and confidence in implementing digital strategy changes [44]. They are increasingly inclined to implement digital transformation strategies to foster the long-term growth of individuals and companies, with the aim of bridging the gap in innovation expectations. Digital transformation may involve fundamental shifts in business processes and innovation trajectories, which requires managers to be highly sensitive and tolerant of new knowledge and technologies [4]. Therefore, if the CEO has a high level of digital literacy when CEO power is exercised to enhance perception of the risk from innovation performance feedback, it can significantly enhance his sensitivity to digital technology and his strategic insight into external trends, allowing him to swiftly convert his expertise and experience into concrete digital strategies and course of action. On the other hand, due to the novelty and cross functionality of digital innovation, enterprises are required to recognize, assimilate, and utilize relevant information regarding digital innovation potential from both internal and external sources in order to initiate digital transformation [45]. CEO digital literacy can effectively identify transformation opportunities, obtain additional information resources, expedite updates to organizational routines and integrate innovative resources based on the enterprise's internal resource advantages. In this circumstance, the CEO could precisely leverage his or her power of strategic decision-making and resource allocation to couple innovative search with digital technology. In view of the above, CEO digital literacy can enhance the confidence of high-power CEOs when there is a gap in innovation expectations, and use their digital knowledge and decision-making authority to accelerate the allocation of resources pertaining to digital transformation. Accordingly, this study proposes.

Hypothesis3a As CEO functional diversity enhances, the positive moderating effect of CEO power on the relationship between innovation expectation gap and enterprise digital transformation will be strengthened.

Hypothesis3b As CEO digital literacy enhances, the positive moderating effect of CEO power on the relationship between innovation expectation gap and enterprise digital transformation will be strengthened.

**Fig. 1:**
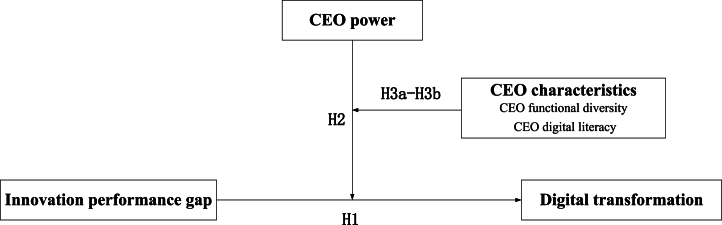
Theoretical model.

The hypothetical path of this study is shown in Fig. 1.

## 3 Methodology and data

### 3.1 Sample and data sources

In the Industry 4.0, its iconic sign is a profound reform that currently strikes the manufacturing industry. Stimulated by the era of digital economy, the traditional capability upgrading is intertwined with development of new capabilities in terms of the manufacturing industry. Hence, digital transformation has become a valuable chance for manufacturing enterprise to revolutionize their technological approach and develop a competitive edge on a worldwide scale. In order to exclude the far-reaching impact of the 2008 financial crisis on the innovation capability of enterprises as well as digital transformation, this paper selects A-share listed companies in the manufacturing industry in the CSMAR database from 2010 to 2021 as the research sample. According to previous research, samples of ST, SST, ∗ST listed companies, and samples with serious missing values and samples of insolvent companies were excluded. All continuous variables were trimmed at the 1 % and 99 % percentiles to mitigate the impact of outliers on the empirical results. Finally, a total of 7244 unbalanced panel data were obtained. In this paper, the digital transformation data are obtained from the annual report of manufacturing enterprises listed on Shanghai and Shenzhen Stock Exchanges. The data of enterprise innovation performance is sourced from the patent information disclosed by China Research Data Service Platform (CNRDS). The marketization index is sourced from the marketization index report of China, which provides statistics on marketization by province. All other financial data is sourced from the CSMAR database.

### 3.2 Variable definition

#### 3.2.1 Dependent variables

Enterprise digital transformation (*Digital*).Strategic management researchers are increasingly relying on tools such as text analysis, natural language processing and machine learning to construct key variables from textual data [46]. Specifically, the Management Discussion and Analysis (MD&A) section of listed company annual reports, owing to its profound insights into a company's operations and the prospects for future development, has emerged as a crucial reference for researchers seeking to grasp corporate strategic trends [24].Therefore, based on the methodologies employed by Zhu et al. [15] and Alkaraan et al. [47], we utilize text analysis and machine learning techniques to extract the frequency of keywords related to digital transformation from the MD&A section of listed company annual reports. First, based on China's national policy semantic system, we construct an enterprise digital terminology dictionary that encompasses both the application of underlying digital technologies and digital technology practices. Second, we employ machine learning technology to conduct in-depth text analysis of the MD&A section, precisely searching and matching digital transformation keywords through Python, and tallying the total frequency of these keywords in each listed company's annual report.To accurately assess the progress of enterprise digital transformation, we also consider the variations in the text length of the MD&A section. We adopt the ratio of the total frequency of digital-related keywords to the length of the MD&A text as an evaluation metric. For ease of expression, we multiplied this metric by 100 to obtain a Digital index. The level of the Digital index directly reflects the degree of a company's positive response to digital transformation strategies.

#### 3.2.2 Independent variable

Innovation expectation gap (*IEG).* The innovation expectation gap reflects the large gap between actual innovation performance (*PA*) and expectation level of innovation (*HA*) [34]. As previous research has shown, patent applications serve as an external measure of the success of innovation, especially in technology-intensive industries [19]. Therefore, the measurement of the actual innovation performance of enterprises is mostly based on the number of patent applications [34]. Compared with utility model patents and design patents, invention patents are characterized by greater difficulty in innovation, higher technical complexity, and higher value to the enterprise innovation system [20]. Therefore, this paper refers to the study by Martínez-Noya and García-Canal [21], which uses the number of invention-based patent applications to measure the actual innovation performance of firms and normalizes the number of invention-based patent applications according to firm size.The measurement of innovation aspirations is based on the behavioral theory of the firm, where enterprises set historical aspirational and social aspirational innovation goals according to their own innovation systems. However, it is difficult for focal enterprises to discerning their social benchmarks due to strategic clusters and the covert characteristics of their industry rivals' innovation systems [48]. For this reason, this study examines how enterprises' historical innovation aspiration goals influence their digital transformation strategies. Its formula is as follows:

$${HA}_{i,t}=\left(1-\gamma_{1}\right){PA}_{i,t-1}+\gamma_{1}{HA}_{i,t-1}$$

In the formula above, the historical innovation aspiration level of an enterprise (${HA}_{i,t}$) in Period t stands for the weighted aggregate of its actual innovation performance (${PA}_{i,t-1}$) in Period t-1 and its historical innovation aspiration target (${HA}_{i,t-1}$) in period t-1; the weight $\gamma_{1}$ is 0.4, with reference to the study made by Chen and Miller [48]. On this basis, truncation is applied to the innovation expectation gap (Firstly, dummy variable $L_{1}$ is set up to measure whether the actual innovation performance is lower than the historical innovation desire level. If ${\;PA}_{i,t}-{HA}_{i,t}<0$, the $L_{1}$ value is set to 1, and otherwise, it's 0; Then, multiply the $L_{1}$ value by (${PA}_{i,t}-{HA}_{i,t}<0$) to obtain a truncated minus variable. Finally, take the absolute value from the minus variable to obtain the final innovation expectation gap (marked as *IEG*); the larger value projects the wider gap between the enterprise's actual innovation performance and the level of historical innovation expectation.

#### 3.2.3 Moderator variables

CEO Power (*CEOPower*). The main sources of CEO power structure include Ownership power, Structural power, Expert power, and Prestige power [49]. In this paper, we refer to Zhang et al. [38] and Lewellyn et al. [50] on the development of comprehensive measures for CEO power, and use principal component analysis to select eight indicators for the above four dimensions to construct an indicator of CEO power, which is referred to as *CEOPower*. Specific indicators are as follows: (1) Ownership power includes the indicator CEO_share, which indicates if the CEO possesses the enterprise's equity. If the condition is true, the value is set to 1, otherwise, it is set to 0. Similarly, if institutional investors hold less enterprise's equity than the industry median, the value of Institute_share is 1; otherwise, it is 0. (2) Structural power encompasses two indicators: Dual, which determines whether the CEO holds the position of both chairman of the board of directors or not; and Insider-director, which determines whether the CEO is an insider director of the enterprise or not. If the condition is true, the value is set to 1, otherwise, it is set to 0. (3) Expert power includes the indicator Rank, which indicates if the CEO holds a senior title. If the CEO has a senior title, the value of the indicator is 1; otherwise, it is 0. And Tenure, refers to whether the CEO's tenure is longer than the median length of service in the industry. If it is longer, the value is 1; otherwise, the value is 0. (4) Prestige power is determined by two indicators: Degree and Part-time job. Degree indicates if the CEO has a master's degree or higher. If the CEO has a master's degree or higher, the value is 1; otherwise, the value is 0. Part-time job indicates whether the CEO works part-time in units other than his own enterprise. If the CEO works part-time in other units, the value is 1; otherwise, the value is 0. We conducted principal component analysis on the above eight indicators, and selected the first principal component as the comprehensive index for measuring the strength of CEO power. The greater the *CEOPower* value, the more power the CEO has.

CEO functional diversity (*CEOFD*). Based on the job classification standards of Crossland et al. [41] and the characteristics of Chinese CEO resumes, this paper categorizes the functional background of CEOs. The specific functions identified include production, research and development, design, human resources, management, marketing, finance, and law. And following Mueller et al. [43], we use the Blau index to calculate CEO functional diversity. This index effectively measures the extent of knowledge that CEOs acquire from various functional areas. The formula for Blau index is given by $Blau=1-\sum_{i=1}^{n}P_{i}^{2}$,where *P*_*i*_ represents the ratio of the CEO's working years in the i-th functional department to the overall working years. Based on the study conducted by Custódio et al. [42], it is assumed that the CEO has an equal number of years of experience in each function due to the absence of specific information regarding the duration of their tenure in the role. The higher the final Blau index indicates that the CEO has more functional experience and a more comprehensive functional knowledge structure.

CEO digital literacy (*CEODT*). Referring to the studies of Firk et al. (2022) [45]and Zhao et al. (2023) [44], we manually retrieve the information of CEO resumes to identify whether CEOs have a digital professional learning or working background in their professional backgrounds. We then construct a dummy variable of CEO digital literacy, *CEODT*. If any of the following conditions are met, the CEO is regarded to possess digital literacy, and CEODT assigns a value of 1; otherwise, it assigns a value of 0. The details are as follows: (1) Here are the specific details: (1) The CEO has a professional background in IT and digitalization, including roles such as “Chief Information Officer”, “Chief Technology Officer”, “Chief Digital Officer” and expertise in areas such as information, computers, software, e-commerce, IT, technology, digital, among others. (2) The CEO's work experience, job positions, or department are related to IT and digital business. (3) The CEO has a digital-related title, such as senior programmer.

#### 3.2.4 Control variables

In order to exclude the interference of other factors, we refer to studies on related topics [20,34], selecting control variables related to firm characteristics, governance structure, industry, and regional characteristics. The control variables are as follows: Size (The natural logarithm of total assets at the end of the period)*Leverage* (Ratio of total liabilities to total assets at the end of the period) *ROA*(net assets income rate)*Asset liquidity*(Ratio of current assets to total assets at the end of period)*Ownership nature* (State-owned enterprises are assigned a value of 1, whereas non-state-owned enterprises are assigned a value of 0) *Board size* (The natural logarithm of the total number of board members)*Executive incentive* (The ratio of management shareholding to total share capital)*Ownership concentration* (The ratio of the largest shareholder to the total share capital) *Industry concentration* (The Herfindahl index is used to measure the market share of all companies in the industry (SIC level 3 code)) *Environmental munificence* (the average sales growth rate over the past five years in the industry in which the business operates (based on SIC level 3 code)) *Marketization* (the natural logarithm of the marketization index of each province in China).

### 3.3 Modeling

To validate the proposed study hypotheses. Initially, all econometric models underwent evaluation and selection by the Hausman test, which demonstrated that the panel fixed effects model had superior performance compared to the random effects model (Prob > chi2 = 0.0000). To address the issue of omitted variables resulting from individual differences and to account for time-varying specific characteristics, all estimate models incorporate person and time two-way fixed effects. Furthermore, the regression models employ Driscoll-Kraay standard errors to address the potential impact of serial correlation, heteroskedasticity, and cross-section correlation in imbalanced panel data on the empirical findings.

## 4 Results

### 4.1 Descriptive statistics and correlation analysis

Table 1 shows the descriptive statistics of 7244 companies, mainly including the mean, standard deviation (SD), Minimum, Median and Maximum values of the variables. As can be seen from Table 1, the mean value of the degree of digital transformation of the sample companies is 0.0388, with a small standard deviation (0.0627), showing a highly concentrated trend with low dispersion. The values of the explanatory variable digital transformation range from 0.0003 to 0.3377, with the overall distribution slightly skewed to the left and containing no zero values. This suggests that most companies are still in the early stages of digital transformation, while very few have achieved a relatively high degree of transformation. Further analyzing the distribution characteristics of the innovation expectation gap, we find that the mean of the innovation expectation gap is 0.0415 with a large standard deviation (0.1164), indicating that the data distribution is more dispersed. In addition, its minimum and median are both 0, while the maximum value reaches 0.6995, which means that the data shows a right-skewed trend. In summary, we can see that there is a large difference between the innovation expectation gap of different companies. The values of the VIF test are also included in Table 1. At the same time, Table 1 also includes the values of the Variance Inflation Factor (VIF) test. Through the VIF test, we found that the VIF values of all variables are not greater than 1.50, with an average value of only 1.17, indicating a low possibility of multicollinearity among variables. According to the correlation coefficient table in Table 2, the correlation coefficients between the core variables are all less than the empirical threshold of 0.5, which further excludes the possible problem of multicollinearity and helps the subsequent regression analysis.

**Table 1:** Descriptive statistics.

Variables	N	Mean	SD	Minimum	Median	Maximum	VIF
*Digital*	7244	0.0388	0.0627	0.0003	0.0136	0.3377	
*IEG*	7244	0.0415	0.1164	0.0000	0.0000	0.6995	1.01
*CEOPower*	7244	0.5030	1.2471	−1.9024	0.3803	3.3012	1.02
*CEOCER*	7244	0.7259	0.0753	0.3784	0.7424	0.8289	1.07
*CEODT*	7244	0.0135	0.1155	0.0000	0.0000	1.0000	1.02
*Size*	7244	22.0641	1.1357	20.0202	21.8955	25.4003	1.50
*Leverage*	7244	0.3944	0.1946	0.0530	0.3847	0.8668	1.39
*ROA*	7244	0.0600	0.1251	−0.6250	0.0623	0.3665	1.03
*Asset liquidity*	7244	0.6346	0.5357	−0.6730	0.6139	2.2245	1.11
*Ownership nature*	7244	0.3029	0.4595	0.0000	0.0000	1.0000	1.34
*Board size*	7244	2.3803	0.2211	1.7918	2.3979	2.9444	1.12
*Executive incentive*	7244	0.1147	0.1697	0.0000	0.0052	0.6360	1.26
*Ownership concentratio*	7244	0.1300	0.1038	0.0087	0.0980	0.5075	1.07
*Industry concentration*	7244	0.7434	0.1824	0.0000	0.7797	0.9594	1.26
*Industry dynamism*	7244	0.0502	0.0368	−0.3282	0.0441	0.1669	1.28
*Marketization*	7244	7.4650	2.1450	0.0100	7.4500	11.4000	1.03

**Table 2:** Correlation matrix.

Variables	*Digital*	*IEG*	3	4	5	6	7	8
1*Digital*	1.0000							
2 *IEG*	−0.0085	1.0000						
3 *CEOPower*	0.1356∗∗∗	0.0469∗∗∗	1.0000					
4 *CEOCER*	−0.0173	0.0109	−0.0203∗	1.0000				
5 *CEODT*	−0.0181	−0.0412∗∗∗	−0.0976∗∗∗	0.0125	1.0000			
6 *Size*	0.0387∗∗∗	−0.0204∗	0.0154	−0.1231∗∗∗	−0.0027	1.0000		
7 *Leverage*	0.0429∗∗∗	−0.0250∗∗	−0.0130	−0.1076∗∗∗	−0.0367∗∗∗	0.4800∗∗∗	1.0000	
8 *ROA*	−0.0238∗∗	−0.0144	0.0077	0.0021	0.0233∗∗	0.0398∗∗∗	−0.0893∗∗∗	1.0000
9 *Asset liquidity*	0.0157	0.0148	0.0178	0.0844∗∗∗	0.0262∗∗	−0.1908∗∗∗	−0.1957∗∗∗	−0.1039∗∗∗
10 *Ownership nature*	0.0509∗∗∗	−0.0061	0.0255∗∗	−0.1937∗∗∗	0.0086	0.3361∗∗∗	0.2502∗∗∗	−0.0209∗
11 *Board size*	0.0290∗∗	0.0137	−0.0040	−0.0169	−0.0096	0.2165∗∗∗	0.1804∗∗∗	−0.0321∗∗∗
12 *Executive incentive*	−0.0657∗∗∗	0.0135	0.0230∗	0.1824∗∗∗	−0.0135	−0.3216∗∗∗	−0.2747∗∗∗	0.0184
13 *Ownership concentratio*	−0.0344∗∗∗	−0.0183	0.0113	−0.0241∗∗	−0.0430∗∗∗	0.1474∗∗∗	0.0158	0.0520∗∗∗
14 *Industry concentration*	−0.0127	0.0029	0.0331∗∗∗	0.0196∗	−0.0078	−0.0045	0.0145	−0.0070
15 *Industry dynamism*	0.0606∗∗∗	−0.0285∗∗	−0.0262∗∗	−0.0500∗∗∗	−0.0179	0.0478∗∗∗	0.0030	0.0112
16 *Marketization*	0.0073	0.0002	0.0478∗∗∗	0.0882∗∗∗	−0.0200∗	0.0854∗∗∗	0.0137	0.0069

Variables	9	10	11	12	13	14	15	16

9 *Asset liquidity*	1.0000							
10 *Ownership nature*	−0.0081	1.0000						
11 *Board size*	0.0085	−0.1505∗∗∗	1.0000					
12 *Executive incentive*	−0.0065	−0.0681∗∗∗	0.2660∗∗∗	1.0000				
13 *Ownership concentratio*	−0.0350∗∗∗	0.1251∗∗∗	−0.3717∗∗∗	−0.1651∗∗∗	1.0000			
14 *Industry concentration*	−0.0282∗∗	−0.1521∗∗∗	0.1438∗∗∗	−0.0271∗∗	−0.0421∗∗∗	1.0000		
15 *Industry dynamism*	0.0127	0.0590∗∗∗	−0.0044	0.0245∗∗	0.0128	0.0560∗∗∗	1.0000	
16 *Marketization*	−0.0114	0.0006	−0.0047	−0.0424∗∗∗	0.0135	−0.0129	−0.0066	−0.0031

Note: ∗, ∗∗ and ∗∗∗ indicate significant correlation at the level of 10 %, 5 % and 1 %, respectively.

### 4.2 Regression result analysis

Prior to conducting the empirical test, the following pre-processing steps were undertaken to enhance the consistency and validity of the estimated model: In the moderating effect test, both the explanatory variables and the moderating variables were centered. Table 3 displays the outcomes of a regression analysis conducted using the stepwise regression approach to examine the correlation between the innovation expectation gap and corporate digital transformation.

**Table 3:** Multiple regression analysis.

Variables	Model (1)	Model (2)	Model (3)	Model (4)	Model (5)	Model (6)
*IEG* (H1)		0.0063∗∗∗	0.0064∗∗∗	0.0064∗∗∗	0.0147∗∗∗	0.0145∗∗∗
		(6.1079)	(5.8320)	(5.7834)	(3.5620)	(3.6914)
*CEOPower*			0.0014∗∗∗	0.0014∗∗∗	0.0016∗∗∗	0.0017∗∗∗
			(8.6391)	(8.6620)	(8.5787)	(9.0853)
*IEG×CEOPower* (H2)			0.0032∗∗	0.0028∗∗	0.0129∗∗∗	0.0127∗∗∗
			(2.3275)	(2.3175)	(3.4693)	(3.4107)
*CEOCER*				0.0035		0.0037
				(0.5042)		(0.5455)
*IEG×CEOCER*				−0.0048		−0.0044
				(-0.1460)		(-0.1346)
*CEOPower×CEOCER*				−0.0080∗		−0.0081∗
				(-2.1181)		(-2.1481)
*IEG×CEOPower×CEOCER*(**H3a**)				0.0767∗∗∗		0.0769∗∗∗
				(4.2840)		(4.3092)
*CEODT*					0.0116∗∗	0.0113∗∗
					(2.8787)	(3.0860)
*IEG×CEODT*					0.6103∗	0.6051∗∗
					(2.2616)	(2.2900)
*CEOPower×CEODT*					0.0160∗	0.0164∗∗
					(2.2426)	(2.3044)
*IEG×CEOPower×CEODT*(**H3b**)					0.7219∗∗	0.7406∗∗
					(2.8643)	(2.9026)
*Size*	0.0001	0.0001	0.0002	0.0002	0.0001	0.0001
	(0.1005)	(0.1198)	(0.1555)	(0.1605)	(0.1058)	(0.1108)
*Leverage*	0.0067∗∗	0.0067∗∗	0.0065∗∗	0.0066∗∗	0.0066∗∗	0.0067∗∗
	(2.5925)	(2.6809)	(2.4616)	(2.4651)	(2.4977)	(2.4988)
*ROA*	0.0006	0.0007	0.0007	0.0007	0.0008	0.0008
	(0.1766)	(0.2223)	(0.2078)	(0.2008)	(0.2261)	(0.2181)
*Asset liquidity*	0.0012∗∗	0.0012∗∗	0.0013∗∗	0.0012∗∗	0.0014∗∗	0.0013∗∗
	(2.2947)	(2.3992)	(2.5175)	(2.2792)	(2.5756)	(2.3469)
*Ownership*	0.0067∗∗∗	0.0066∗∗∗	0.0065∗∗	0.0065∗∗	0.0065∗∗	0.0065∗∗
	(3.3590)	(3.3595)	(3.2178)	(3.2165)	(3.2221)	(3.2242)
*Board size*	−0.0031	−0.0032	−0.0031	−0.0033	−0.0030	−0.0032
	(-1.5017)	(-1.5076)	(-1.4879)	(-1.5932)	(-1.4389)	(-1.5437)
*Executive incentive*	0.0127∗∗∗	0.0125∗∗∗	0.0125∗∗∗	0.0121∗∗∗	0.0124∗∗∗	0.0121∗∗∗
	(4.4316)	(4.3585)	(4.1318)	(4.2364)	(4.1977)	(4.2969)
*Ownership concentration*	0.0159∗∗	0.0158∗∗	0.0150∗∗	0.0147∗∗	0.0152∗∗	0.0150∗∗
	(3.0661)	(3.0249)	(2.8758)	(2.7161)	(2.8285)	(2.6745)
*Industry concentration*	0.0091∗∗	0.0091∗∗	0.0090∗∗	0.0093∗∗	0.0091∗∗	0.0093∗∗
	(2.9459)	(2.9141)	(2.8347)	(2.8279)	(2.7260)	(2.7191)
*Environmental munificence*	−0.0167	−0.0164	−0.0169	−0.0161	−0.0168	−0.0160
	(-1.5726)	(-1.5691)	(-1.6158)	(-1.5080)	(-1.6096)	(-1.5010)
*Marketization*	−0.0030∗∗	−0.0030∗∗	−0.0029∗∗	−0.0029∗∗	−0.0029∗∗	−0.0028∗∗
	(-2.7608)	(-2.7538)	(-2.6996)	(-2.7032)	(-2.6344)	(-2.6353)
*Constant*	0.0351	0.0347	0.0340	0.0341	0.0348	0.0349
	(1.4500)	(1.4292)	(1.3908)	(1.3666)	(1.4341)	(1.4090)
*Firm fixed effects*	Yes	Yes	Yes	Yes	Yes	Yes
*Year fixed effects*	Yes	Yes	Yes	Yes	Yes	Yes

*Observations*	7244	7244	7244	7244	7244	7244
*F*	118.1201	131.3963	1.1e+03	221.7505	62.8896	87.6734
*R2*	0.0943	0.0950	0.0962	0.0975	0.0967	0.0980

Note:∗∗∗, ∗∗, and ∗ represent significance levels at 1 %, 5 %, and 10 %, respectively; t-values are in parentheses, and the above models are adjusted for Driscoll-Kraay standard errors.

Table 3 Model (1) is the base model containing only control variables. Model (2) adds Innovation expectation gap (*IEG*) to model (1), which has a significantly positive regression coefficient (*β* = 0.0063, *p* < 0.01) and the regression results for this variable remain robust in subsequent models. From the estimation results, for every 1 unit standard deviation increase in the firm's innovation expectation gap, the proportion of the firm's digital transformation word frequency in the annual report text will increase by 0.07 % (=0.063 × 0.1164), which can explain 1.89 % of the sample mean of 0.0388 (=0.07 %/0.0388), and has a certain degree of economic significance.The above results indicate that as the innovation expectation gap widens, enterprises' motivation to reverse the current problem of poor innovation performance through digital transformation increases, i.e., the innovation expectation gap has a positive impact on enterprises' digital transformation, and H1 is supported.

Model (3) in Table 3 lists the regression results of the moderating effect of CEO power on the gap between innovation expectation and enterprise digital transformation. According to the results of model (3), the cross term of innovation expectation gap (*IEG*) and CEO power (*CEOPower*) (*IEG×CEOPower*) shows a significant positive correlation with enterprise digital transformation (*Digital*) (*β* = 0.0032, *p* < 0.05), and remains robust in the subsequent model. This indicates that with the increase of CEO power, the positive impact of innovation expectation gap on enterprise digital transformation will be enhanced, and H2 is supported.

Model (4) and model (5) in Table 3 list the test results of the secondary moderating effect of CEO functional diversity and CEO digital literacy respectively. According to the results of model (4), the interaction terms of innovation expectation gap (*IEG*), CEO power (*CEOPower*) and CEO functional diversity (*CEOFD*) (*IEG×CEOPower×CEOFD*) and enterprise Digital transformation (*Digital*) are significantly positive (*β* = 0.0767, *p* < 0.01) and remained robust in subsequent models. This indicates that CEO functional diversity enhances the promoting effect of CEO power on the positive relationship between innovation expectation gap and enterprise digital transformation, assuming that H3a is supported. According to the results of model (5), the interaction terms of innovation expectation gap (*IEG*), CEO power (*CEOPower*) and CEO Digital literacy (*CEODT*) (*IEG×CEOPower×CEODT*) and enterprise digital transformation (*Digital*) are significantly positive (*β* = 0.7219, *p* < 0.05),and it remains robust in subsequent models. This indicates that CEO digital literacy enhances the promoting effect of CEO power on the positive relationship between innovation expectation gap and enterprise digital transformation, assuming that H3b is supported.

### 4.3 Robustness tests

#### 4.3.1 Propensity score matching (PSM) method

To enhance the accuracy of the estimation results, the treatment group (with a significant gap in innovation expectations) and the control group (with a minimal gap in innovation expectations) underwent Propensity Score Matching (PSM) using radius matching technique. Next, we analyze the disparities between the treatment group and the control group in the execution of the corporate digital transformation plan while ensuring that the covariates remain constant. This is done to minimize the influence of selection bias and confounding variables. The precise procedure is as follows: The first step involves defining the dummy variable for the group with a significant difference in innovation expectations. This variable, known as the processing variable, is set to 1 when the innovation expectation gap is larger than the median, and 0 when it is smaller than the median. Additionally, the enterprise digital transformation is defined as the outcome variable. Simultaneously, a set of control factors, such as enterprise characteristics, governance structure, industry, and area described before, are incorporated as covariates. Furthermore, we employ the aforementioned covariates to estimate the processing variables using Logit regression, yielding the propensity score value. We next opt for the radius matching approach. The findings indicate that the magnitude of the standardized deviation of the matched variables does not surpass 5 %, and the P statistics of the T-test do not exceed 10 %. This indicates that there was no substantial disparity between the variables in the treatment and control groups. Furthermore, there is a minimal loss of samples within the typical value range, resulting in a satisfactory overall matching outcome. Based on the matched samples, the influence of the disparity in innovation expectations on the digital transformation of enterprises is reassessed. The findings are displayed in Table 4. Upon mitigating any selection bias and confounding variables, the pertinent research findings of this paper have remained mostly unchanged.

**Table 4:** PSM method.

Variables	Model (1)	Model (2)	Model (3)	Model (4)	Model (5)
*IEG* (H1)	0.0063∗∗∗	0.0064∗∗∗	0.0063∗∗∗	0.0146∗∗∗	0.0145∗∗∗
	(6.0989)	(5.8229)	(5.7754)	(3.5560)	(3.6846)
*CEOPower*		0.0014∗∗∗	0.0014∗∗∗	0.0016∗∗∗	0.0016∗∗∗
		(8.8735)	(8.9870)	(8.6555)	(9.2969)
*IEG×CEOPower* (H2)		0.0032∗∗	0.0028∗∗	0.0128∗∗∗	0.0126∗∗∗
		(2.3566)	(2.3497)	(3.3889)	(3.3350)
*CEOCER*			0.0034		0.0036
			(0.4825)		(0.5231)
*IEG×CEOCER*			−0.0046		−0.0043
			(-0.1430)		(-0.1316)
*CEOPower×CEOCER*			−0.0078∗		−0.0079∗
			(-2.1151)		(-2.1461)
*IEG×CEOPower×CEOCER*(**H3a**)			0.0763∗∗∗		0.0765∗∗∗
			(4.1799)		(4.2042)
*CEODT*				0.0116∗∗	0.0113∗∗
				(2.8663)	(3.0788)
*IEG×CEODT*				0.6098∗	0.6048∗∗
				(2.2577)	(2.2871)
*CEOPower×CEODT*				0.0157∗	0.0161∗
				(2.1407)	(2.2044)
*IEG×CEOPower×CEODT*(**H3b**)				0.7148∗∗	0.7335∗∗
				(2.7835)	(2.8222)
*Controls*	Yes	Yes	Yes	Yes	Yes
*Constant*	0.0347	0.0340	0.0342	0.0348	0.0349
	(1.4293)	(1.3904)	(1.3679)	(1.4335)	(1.4103)
*Firm fixed effects*	Yes	Yes	Yes	Yes	Yes
*Year fixed effects*	Yes	Yes	Yes	Yes	Yes

*Observations*	7227	7227	7227	7227	7227
*F*	113.0249	62.4818	191.3822	60.6596	87.5745
*R2*	0.0946	0.0958	0.0971	0.0963	0.0976

#### 4.3.2 Two-stage Heckman selection model

Since the disclosure of information about the digital transformation of enterprises can convey their core technology to the outside world. In order to prevent technology leakage or other factors, some enterprises may not force the disclosure of information related to digital transformation, and this paper assigns the value of not announcing the digital transformation vocabulary to 0 during data processing, which may cause some sample selection bias. Therefore, this paper uses the Heckman two-stage estimation method to mitigate this endogeneity problem. In the first stage regression, whether the company disclosed digital transformation related characteristic words (*Digital_DUM*) was selected as the explanatory variable, and *Size*、*Leverage*、*ROA*、*Asset liquidity、Ownership nature*、*Board size*、*Executive incentive*、*Ownership concentratio*、*Industry concentration*、*Environmental munificence*、*Marketization* as explanatory variables. At the same time, the mean of the digital transformation level of other enterprises in the same region and industry in the same year (*Digital_IV*) is used as an exclusive explanatory variable. This is because enterprises ' digital transformation disclosures are influenced by institutional pressures in the same region and in the same industry [51]. On this basis, the probit model is constructed for regression to obtain the Inverse Mills Ratio (*IMR*). The results of the first stage regression are shown in Table 5 Model (1), the regression coefficients of the excluded explanatory variables are significantly positive, which is in line with our theoretical expectations. In the second stage regression analysis, *IMR* is introduced into the model as a control variable, and the results are shown in models (2) to (5) in Table 5.The coefficient of *IMR* is insignificant, indicating that the sample selection bias problem in the original equation is not significant. Meanwhile, the core explanatory variables still have a significant positive impact in the second stage, and their effect sizes are not significantly different from the benchmark regression. In summary, the benchmark regression results are credible when sample selection bias is taken into account.

**Table 5:** Two-stage Heckman selection model.

Variables	Model (1)	Model (2)	Model (3)	Model (4)	Model (5)	Model (6)
*Digital_IV*	14.4939∗∗∗					
	(4.3160)					
*IEG* (H1)		0.0063∗∗∗	0.0043∗∗∗	0.0042∗∗	0.0126∗∗	0.0124∗∗
		(6.0897)	(3.2541)	(3.1400)	(2.7861)	(2.7888)
*CEOPower*			0.0015∗∗∗	0.0015∗∗∗	0.0017∗∗∗	0.0017∗∗∗
			(8.9448)	(8.8586)	(8.8826)	(9.2675)
*IEG×CEOPower* (H2)			0.0024∗∗	0.0023∗∗	0.0121∗∗	0.0122∗∗
			(2.3118)	(2.4270)	(3.1681)	(3.1305)
*CEOCER*				0.0041		0.0043
				(0.5856)		(0.6271)
*IEG×CEOCER*				−0.0191		−0.0189
				(-1.1868)		(-1.1680)
*CEOPower×CEOCER*				−0.0075∗		−0.0076∗
				(-1.8662)		(-1.8928)
*IEG×CEOPower×CEOCER*(**H3a**)				0.0413∗∗		0.0414∗∗
				(2.4936)		(2.5022)
*CEODT*					0.0103∗∗	0.0099∗∗
					(2.9645)	(3.2156)
*IEG×CEODT*					0.6104∗∗	0.6028∗∗
					(2.2680)	(2.2980)
*CEOPower×CEODT*					0.0144∗	0.0147∗∗
					(2.1999)	(2.2686)
*IEG×CEOPower×CEODT*(**H3b**)					0.7207∗∗	0.7347∗∗
					(2.8855)	(2.9283)
*IMR*		−0.0509	−0.0520	−0.0538	−0.0520	−0.0537
		(-1.3215)	(-1.3404)	(-1.3240)	(-1.3376)	(-1.3194)
*Controls*	Yes	Yes	Yes	Yes	Yes	Yes
*Constant*	2.5203	0.0348	0.0341	0.0336	0.0349	0.0344
	(1.2381)	(1.4232)	(1.3841)	(1.3422)	(1.4257)	(1.3829)
*Firm fixed effects*	Yes	Yes	Yes	Yes	Yes	Yes
*Year fixed effects*	Yes	Yes	Yes	Yes	Yes	Yes

*Observations*	7244	6474	6474	6474	6474	6474
*Wald chi2/F*	61.83	143.6825	121.7232	27.2994	36.4389	176.6501
*PseudoR2/R2*	0.6430	0.0951	0.0962	0.0972	0.0967	0.0977

#### 4.3.3 Replacing the dependent variable

According to Jafari-Sadegh et al. [52], the availability of information and communication technologies (ICTs) and their integration to facilitate business process connectivity may contribute to the continued development of digital technologies within manufacturing companies. Therefore, we use Li et al.'s [53]methodology to measure enterprise digital transformation by incorporating digital investment levels. Digital investment includes both hardware investments, such as computers and electronic equipment, and software investments, such as software assets. The digital investment level is measured as the proportion of digital investments in the company's net asset value. We have constructed a comprehensive alternative indicator for digital transformation by standardizing the word frequency index and digital investment level. As shown in Table 6, the introduction of this indicator did not change the results of the hypothesis verification, further confirming the validity and reliability of this study.

**Table 6:** Replacing the dependent variable.

Variables	Model (1)	Model (2)	Model (3)	Model (4)	Model (5)
*IEG* (H1)	0.0849∗	0.0914∗∗	0.0913∗∗	0.2107∗∗	0.2057∗∗∗
	(2.0180)	(2.4265)	(2.4078)	(3.2482)	(3.3205)
*CEOPower*		−0.0047	−0.0047	−0.0016	−0.0015
		(-0.2637)	(-0.2700)	(-0.0862)	(-0.0806)
*IEG×CEOPower* (H2)		0.1071∗∗	0.0996∗	0.2512∗∗∗	0.2510∗∗∗
		(2.3079)	(2.1229)	(3.9981)	(3.7544)
*CEOCER*			0.2820		0.2867
			(1.7522)		(1.7707)
*IEG×CEOCER*			0.0002		0.0063
			(0.0003)		(0.0092)
*CEOPower×CEOCER*			−0.2541∗		−0.2560∗
			(-1.9475)		(-1.9559)
*IEG×CEOPower×CEOCER*(**H3a**)			1.3763∗∗		1.3804∗∗
			(2.5079)		(2.5204)
*CEODT*				0.1189∗∗	0.0968∗∗
				(2.5721)	(2.3891)
*IEG×CEODT*				8.8241∗∗	8.4580∗∗
				(2.4165)	(2.3743)
*CEOPower×CEODT*				0.1979	0.2063
				(1.4993)	(1.5377)
*IEG×CEOPower×CEODT*(**H3b**)				10.7146∗∗	11.2698∗∗
				(2.4614)	(2.5676)
*Controls*	Yes	Yes	Yes	Yes	Yes
*Constant*	1.6846	1.6900	1.6793	1.7017	1.6914
	(1.7553)	(1.7448)	(1.7546)	(1.7595)	(1.7696)
*Firm fixed effects*	Yes	Yes	Yes	Yes	Yes
*Year fixed effects*	Yes	Yes	Yes	Yes	Yes

*Observations*	7244	7244	7244	7244	7244
*F*	1.3e+03	563.3477	83.8949	167.7845	76.6353
*R2*	0.0225	0.0226	0.0232	0.0227	0.0233

#### 4.3.4 Excluding the interference of strategic information disclosure

Although, the indicators of the degree of digital transformation of enterprises constructed by textual analysis can theoretically reflect the actual status of digital transformation of enterprises in a more objective way, their validity is inevitably limited by the potential bias of enterprises' strategic information disclosure [54]. Specifically, some enterprises may strategically exaggerate the effectiveness of their digital transformation in their annual reports and other public documents due to strategic considerations, such as attracting investors' attention and creating a positive market image, thereby misleading investors and the public. Conversely, other companies may deliberately reduce or obfuscate the disclosure of information related to digital transformation for reasons such as protecting strategic secrets and avoiding competitive disadvantages. Due to these phenomena, our digital transformation indicators may not be able to comprehensively and accurately portray the real situation of a enterprises' digital transformation, which in turn affects the accuracy and reliability of the assessment. Therefore, we further exclude potential strategic disclosure interference to enhance the robustness and reliability of our analytical conclusions. First, some samples are excluded, i.e., those that have been penalized by the China Securities Regulatory Commission or stock exchanges for disclosure of information and other issues. Second, only the sample of listed companies with excellent or good disclosure assessment results is retained, as such companies are less likely to engage in strategic disclosure. Finally, market service organizations represented by auditors and analysts are added as a control variable because they can actively play the function of information intermediary and increase the transparency of the company based on a large amount of information collection and field research work [55]. In particular, the *Big4* dummy variable is set based on whether the company has hired an international Big 4 accounting firm, which in turn evaluates the quality of the company's external audit. In addition, the number of securities analysts focusing on the same listed company is used to measure the degree of analyst focus, denoted as *Analyst*.The empirical results are presented in Table 7, and all the conclusions still hold. It can be seen that all the research hypotheses of this paper are still supported by empirical evidence after excluding the effect of strategic disclosure of listed companies.

**Table 7:** Excluding the interference of strategic information disclosure.

Variables	Model (1)	Model (2)	Model (3)	Model (4)	Model (5)
*IEG* (H1)	0.0048∗∗∗	0.0049∗∗∗	0.0051∗∗∗	0.0152∗∗∗	0.0155∗∗∗
	(4.5424)	(4.3552)	(4.3661)	(4.1759)	(4.3029)
*CEOPower*		0.0011∗∗∗	0.0011∗∗∗	0.0013∗∗∗	0.0013∗∗∗
		(3.3797)	(3.4917)	(4.5765)	(4.8180)
*IEG×CEOPower* (H2)		0.0043∗∗	0.0040∗∗	0.0151∗∗∗	0.0150∗∗∗
		(2.9166)	(2.9918)	(3.7174)	(3.6505)
*CEOCER*			−0.0056		−0.0050
			(-0.7553)		(-0.6844)
*IEG×CEOCER*			0.0249		0.0256
			(0.7351)		(0.7481)
*CEOPower×CEOCER*			−0.0032		−0.0034
			(-0.8979)		(-0.9458)
*IEG×CEOPower×CEOCER*(**H3a**)			0.0632∗∗∗		0.0634∗∗∗
			(4.2467)		(4.2214)
*CEODT*				0.0130∗∗∗	0.0134∗∗∗
				(4.2970)	(4.5139)
*IEG×CEODT*				0.7627∗∗∗	0.7721∗∗∗
				(3.7488)	(3.7907)
*CEOPower×CEODT*				0.0155∗	0.0158∗
				(1.9026)	(1.9436)
*IEG×CEOPower×CEODT*(**H3b**)				0.8045∗∗∗	0.8121∗∗∗
				(3.2352)	(3.2211)
*Big4*	0.0009	0.0010	0.0011	0.0009	0.0011
	(0.5247)	(0.5578)	(0.6651)	(0.5499)	(0.6509)
*Analyst*	0.0011∗∗	0.0011∗∗	0.0011∗∗	0.0011∗∗	0.0011∗∗
	(2.8506)	(2.7624)	(2.6631)	(2.7389)	(2.6384)
*Controls*	Yes	Yes	Yes	Yes	Yes
*Constant*	0.0272	0.0266	0.0274	0.0276	0.0284
	(1.1367)	(1.1034)	(1.0926)	(1.1402)	(1.1271)
*Firm fixed effects*	Yes	Yes	Yes	Yes	Yes
*Year fixed effects*	Yes	Yes	Yes	Yes	Yes

*Observations*	6378	6378	6378	6378	6378
*F*	164.8732	237.8169	52.9011	51.1494	53.9331
*R2*	0.0960	0.0971	0.0980	0.0978	0.0987

#### 4.3.5 Controlling for omitted variables

On the one hand, R&D investment is a key element in the continuous iteration of enterprises' digital technologies. High-intensity R&D investment can support enterprises to adopt advanced technologies, develop new products, optimize production processes, and make data-driven decisions, which can significantly improve enterprises' digitalization level [56]. On the other hand, abundant capital can lead to the cost pressure of digital transformation. Digital transformation is not just a digital iteration of technology or equipment, but a deep industrial restructuring that cuts across modules such as organizational processes, business models, operational management, and business models [11].The investment in information technology construction and ICT R&D in the early stage of transformation will put tremendous pressure on the established cost structure. In addition, the digital transformation of enterprises may lead to the innovation of new products, technologies, or business models, which may generate a large number of intellectual property rights such as patents, trademarks, and brands. However, these intellectual property rights can easily lead to technological spillover effects or infringement [57]. Strengthening the protection of intellectual property rights can effectively crack down on IPR infringement and ensure that enterprises benefit from the fruits of digital technology, thereby accelerating the process of digital transformation of enterprises.

Next, we include R&D intensity (measured by the ratio of R&D expenditures to total assets, denoted as *R&D intensity*), financing constraints (measured by the SA index, denoted as *SA*), and intellectual property protection (the ratio of patents that are not infringed in the region where the firms are located, denoted as *IPP*) in the control variables and re-run the regression analysis. The empirical results are shown in Table 8, we find that the regression coefficient of R&D investment intensity is not significant (*β* = 0.0006, *p* > 0.1), the regression coefficients of financing constraints are all significantly negative (*β* = −0.0242, *p* < 0.01), and the regression coefficient of intellectual property rights protection is significantly positive (*β* = 0.0084, *p* < 0.01), and the above results are consistent in the subsequent models. Meanwhile, after controlling for potential omitted variables, all the hypotheses of this paper are still supported.In summary, the finding that R&D investment intensity alone may not be the key driver of digital transformation is rooted in the complex and multidimensional nature of digital transformation [11]. Digital transformation involves not only technological innovation, but also corporate strategy, organizational structure, business processes, and cultural transformation. Although R&D investment can accelerate technological innovation and product iteration, it lacks synergy with other transformation elements and is difficult to drive transformation success on its own. What's more, the success of digital transformation depends on the support of the external environment, of which optimizing the financing environment and improving the intellectual property protection system are particularly critical. An improved financing environment ensures that enterprises have sufficient financial resources to support long-term digital transformation investments [58], while a sound intellectual property protection system provides legal protection for enterprise innovation and stimulates enterprise innovation vitality.

**Table 8:** Controlling for omitted variables.

Variables	Model (1)	Model (2)	Model (3)	Model (4)	Model (5)
*IEG* (H1)	0.0056∗∗∗	0.0058∗∗∗	0.0057∗∗∗	0.0134∗∗	0.0133∗∗∗
	(5.9410)	(5.4635)	(5.4190)	(3.2433)	(3.3519)
*CEOPower*		0.0014∗∗∗	0.0014∗∗∗	0.0019∗∗∗	0.0019∗∗∗
		(8.9670)	(9.1879)	(7.3913)	(7.9588)
*IEG×CEOPower* (H2)		0.0039∗∗	0.0034∗∗	0.0188∗∗	0.0186∗∗
		(2.9338)	(2.9347)	(3.1602)	(3.1029)
*CEOCER*			0.0027		0.0030
			(0.4046)		(0.4439)
*IEG×CEOCER*			−0.0030		−0.0027
			(-0.0918)		(-0.0835)
*CEOPower×CEOCER*			−0.0084∗		−0.0085∗
			(-2.1600)		(-2.1949)
*IEG×CEOPower×CEOCER*(**H3a**)			0.0741∗∗∗		0.0743∗∗∗
			(4.1859)		(4.2178)
*CEODT*				0.0107∗∗	0.0104∗∗
				(2.7492)	(2.9577)
*IEG×CEODT*				0.5647∗	0.5610∗
				(2.1020)	(2.1297)
*CEOPower×CEODT*				0.0331∗∗	0.0335∗∗
				(2.3349)	(2.3723)
*IEG×CEOPower×CEODT*(**H3b**)				1.1093∗∗	1.1271∗∗
				(2.6864)	(2.7186)
*R&D intensity*	0.0006	0.0005	0.0007	0.0005	0.0007
	(0.1533)	(0.1438)	(0.1850)	(0.1365)	(0.1793)
*SA*	−0.0242∗∗∗	−0.0245∗∗∗	−0.0244∗∗∗	−0.0246∗∗∗	−0.0245∗∗∗
	(-7.1393)	(-7.4087)	(-7.2832)	(-7.4308)	(-7.3168)
*IPP*	0.0084∗∗∗	0.0085∗∗∗	0.0086∗∗∗	0.0086∗∗∗	0.0087∗∗∗
	(6.9594)	(6.7397)	(6.7714)	(6.8613)	(6.9195)
*Controls*	Yes	Yes	Yes	Yes	Yes
*Constant*	−0.0565∗∗	−0.0583∗∗	−0.0578∗∗	−0.0571∗∗	−0.0566∗∗
	(-2.6284)	(-2.6903)	(-2.6718)	(-2.5991)	(-2.5787)
*Firm fixed effects*	Yes	Yes	Yes	Yes	Yes
*Year fixed effects*	Yes	Yes	Yes	Yes	Yes

*Observations*	7244	7244	7244	7244	7244
*F*	169.5900	688.7781	81.8490	378.6577	248.2280
*R2*	0.1022	0.1037	0.1049	0.1042	0.1055

## 5 Discussion and conclusions

In practice, such impasses as weak independent innovation and external constraints from technical “bottlenecks” still currently afflict the manufacturing industry of China. Therefore, the innovation-driven development of the manufacturing industry at a high level is the key to help China shake off its public image as a “big but not major country” in the manufacturing industry. Although a large number of studies have explored the antecedents that affect the innovation performance of manufacturing enterprises, there is still a lack of study on what strategic responses enterprises should adopt to improve their underperforming innovation. Our data confirms the behavioral model of how manufacturing enterprises embrace digital transformation as a strategic reaction, influenced by feedback on innovation performance. First, aligned with the behavioral theory of the firm, research has found that if enterprises fail to fulfill their innovation objectives, they will proactively engage in search behavior to improve their innovation capabilities through digital transformation strategies. As Zhuo and Chen [5] pointed out, digital transformation can solve innovation dilemmas by improving the quality of innovation and enhancing the capabilities to adopt and adapt technology. Second, as enhancement is made in the power of a CEO-the digital strategic decision-maker and the main character in performance feedback, the ensuing impacts will be imposed upon rationality of executive's decisions, and furthermore, some effects therefrom will act on executive's non-rational perception [18]. Research has shown that the greater the power a CEO possesses, the more it can stimulate the CEO's ability to take risks and allocate resources. Consequently, this strengthens the CEO's motivation to implement digital transformation strategies in order to address the gap in innovation expectations. Finally, when the CEO has a range of professional backgrounds and is skilled in digital technologies, the CEO has the higher information and management advantages in the professional field. This advantage enables the CEO to mitigate the uncertainty in both internal and external environments, while bolstering his/her confidence and expertise in making risk-related decisions. Therefore, CEO power has a greater impact on strengthening the relationship between the innovation expectation gap and enterprise digital transformation.

### 5.1 Implications for theory

This study makes the following contributions to the literature on enterprise digital transformation and the behavioral theory of the firm.

First, from the perspective of the behavioral theory of the firm, this study explores the triggering mechanism of innovation expectation gaps on enterprise digital transformation strategies. It enhances the understanding of the antecedents of digital transformation, and expands the research scope of the behavioral theory of the firm. Distinguishing from existing research on enterprise digital transformation strategic decisions that relies on institutional theory, resource-based view, dynamic capabilities, and upper echelon theory [11,14], this study utilizes the analytical framework of the behavioral theory of the firm to deeply explore the decision-making process of decision-makers within the context of bounded rationality. By verifying the phenomenon of manufacturing enterprises setting innovation reference points and forming desired aspirations based on aspiration goals, this study indicates that when innovation performance does not meet expectations, enterprises tend to expand their search for innovation through digital strategic innovation. This study not only provides an innovative theoretical perspective for understanding enterprise digital transformation strategies, but also enriches the exploratory research on the antecedents of digital transformation. Additionally, it provides valuable insights for the strategic analysis of digital transformation at the micro-enterprise level. Furthermore, this study applies the analytical framework of the behavioral theory of the firm to non-financial performance, particularly the innovation performance of manufacturing enterprises. In manufacturing firms, innovation goals often take precedence over financial goals, and are crucial for maintaining competitiveness. These enterprises strive to keep their innovation activities on the right performance track [34]. This study echoes the viewpoint of Martínez-Noya and García-Canal [21], who advocate extending the behavioral theory of the firm to encompass organizational goals and aspirations beyond mere financial performance. By thoroughly examining the impact of innovation performance feedback on strategic decisions for enterprise digital transformation, this study supplements the limited literature on this topic and clarifies the logical relationship between innovation aspiration goals and related strategic decisions.

Second, we incorporate CEO power into the performance feedback decision-making model to examine its impact on the relationship between innovation performance gaps and enterprise digital transformation, building upon the findings of Marabelli and Galliers [59] that highlight the role of top management team power in driving or altering the speed of digital transformation. This provides new insights and advancements regarding the conflict between CEO power and performance feedback within the framework of the behavioral theory of the firm. Empirical studies provide inconsistent evidence on problem search effects on search results in various organizations [18]. This empirical controversy may arise from the fact that most studies focus on problem search as a process carried out by the enterprise at a general level, assuming it to be a routine and automatic process, without considering the potential influence of the CEO in this process [28]. Although previous scholars have examined the moderating influence of CEO tenure [60] and CEO's organizational recognition [61] on the relationship between performance feedback and decision-making behavior. However, as the CEO is responsible for making decisions on innovation performance feedback, how the CEO power acts on the relationship between behavioral driving force and organizational decision-making through risk-taking and resource allocation remains an unresolved issue in prior studies. This study analyses the role of CEO power in regulating the relationship between innovation performance feedback and enterprise digital transformation, highlighting the boundary conditions for innovation expectation gaps to trigger enterprise digital transformation decisions. From the perspective the leading role of decision makers, it enriches the mechanism of situations where CEO power acts on the innovation search process. In view of the above, it not only contributes to the conversation about CEO power and innovation performance feedback in the context of the behavioral theory of the firm, but also further enriches the upper echelons theory in terms of expanded application of CEO power in digital transformation strategies.

Third, this study integrates CEO traits (i.e., CEO functional diversity and CEO digital literacy) into the analytical framework, enhances and broadens the scope of research on CEO traits in innovation performance feedback and digital transformation. Performance feedback triggers problem search. In this regard, however, it's complicated to erect the micro perception foundation and set up pertinent goals [62]. Despite that a CEO acts as a main character in those decisions triggering problem search, the mechanism how CEO power allocation is intertwined with intrinsic traits to push forward search behaviors of enterprises has not yet been clearly identified. Even both risk taking and resource allocation for decisions could be consolidated with CEO power, those enterprises with intention of digital transformation are extremely destitute of the necessary knowledge hierarchy in vertical fields for commencement of digital transformation [45]. According to this paper, the response behaviors of digital transformation triggered by CEO power from the perspective of innovation performance feedback will be influenced by diverse CEO functions and differences in CEO traits like digital literacy. In other words, a CEO may be driven by diverse CEO functions and digital literacy to allocate CEO power to the specific action innovative search (that is, digital transformation of enterprises). This issue reinforces and expands upon the perspective presented by Blagoeva et al.'s [18] that CEO power will affect performance feedback and behavioral results, and contributes to a deeper and more comprehensive understanding of the micro individual foundation about the process that innovation performance feedback triggers problem search. In particular, it clarifies the interlocking mechanism between CEO power and CEO traits, and enriches researches about digital transformation of enterprises, in terms of complexity in micro mechanisms.

### 5.2 Implications for practice

This study has certain practical significance: Firstly, decision makers in manufacturing enterprises should consider the innovation ambition objective as the standard for evaluating innovation. This is crucial for accurately determining the success or failure of innovation. Manufacturing enterprises should proactively select innovation targets based on their own development and progress. Under the supervision of innovation performance feedback, enterprises should continually improve their own capacity for innovation and consistently address the deficiencies encountered during the innovation process. When the innovation performance is significantly lower than expected, it becomes even more crucial to initiate a search for problems and assist enterprises in expediting the establishment of an efficient, collaborative, and open innovation system through the empowerment of digital technology. This will enable them to take advantages of the opportunities presented by technological and industrial revolutions and reconstruct their competitive advantages. Secondly, it is essential for enterprises to establish an organizational framework based on trust and proper delegation of authority. By granting CEOs the necessary decision-making power and autonomy, key human resources can be fully utilized for their innovative and proactive abilities. To address the present innovation issue through digital transformation, an enterprise must leverage the CEO's core competence to forecast value and utilize the CEO's resource allocation methods to strategically plan and implement changes inside the organization. By updating digital technology, the building of enterprise innovation system may be more effectively facilitated. Thirdly, the findings provide practical insights for companies to explore organizational and talent breakthroughs under digital transformation. Digital technologies are continually breaking down organizational boundaries due to their generativity, forgeability, and combinability. The initiation and implementation of digital strategies are therefore extremely challenging and require strong knowledge integration capabilities of decision makers [45]. Enterprise digital strategy is more than the iterative upgrading of digital technology, it requires the CEO to provide proper guidance in the modules of strategy, process, business model, organizational model and culture construction. Therefore, enterprises should have the support of compound CEOs with key skills related to digitalization.

### 5.3 Limitations and future research

This article may have the following constraints. On the one hand, it is both the external institutional environment as well as the internal resource endowment that influence how an enterprise formulates and implements its strategic decisions. The impact of innovation performance feedback on the intensity of strategic change may be closely related to factors such as institutional environment and resources [26]. Further research can examine the boundary conditions under which the innovation expectation gap triggers enterprises' digital transformation decisions in light of the unique institutional environment of China's transitioning economy as well as the internal resources of the enterprises. This provides Chinese manufacturing enterprises with valuable policy insights for driving innovation and development through digital empowerment. On the other hand, enterprises evaluate performance by considering multiple reference points simultaneously. However, these reference points may not be consistent in indicating success or failure. Thus, In response to inconsistent feedback, decision makers use two distinct decision rules: problem-solving and self-enhancement [18]. Follow-up research can explore the strategic response behavior of enterprises under the perspective of inconsistent positive feedback and negative performance prospects of innovation performance, and promote the relevant literature on inconsistent feedback and decision rules.

## CRediT authorship contribution statement

**Dongwei Li:** Writing – review & editing, Writing – original draft, Visualization, Validation, Software, Resources, Methodology, Investigation, Formal analysis, Data curation, Conceptualization. **Jaffar Abbas:** Visualization, Validation, Supervision, Software, Resources, Methodology, Investigation, Data curation, Conceptualization. **Haojun Wang:** Writing – review & editing, Writing – original draft, Visualization, Validation, Software, Resources, Methodology, Investigation, Formal analysis, Data curation, Conceptualization. **Khalid Al-Sulaiti:** Visualization, Validation, Software, Resources, Methodology, Investigation, Data curation, Conceptualization. **Bei Lyu:** Writing – review & editing, Writing – original draft, Visualization, Validation, Supervision, Software, Resources, Project administration, Methodology, Investigation, Funding acquisition, Formal analysis, Data curation, Conceptualization.

## Funding

Guizhou Province University Humanities and Social Sciences Research Funding Project of Guizhou Province Universities in China (No.2024RW138), Guizhou Minzu University Fund Project in China (No. GZMZSK[2022]YB11), National Social Science Fund Project of China (No.23BMZ055), Major Program of Anhui University Scientific Research Project in China (No.2023AH040053), Action Program for Young-Middle aged Teachers Training in Anhui Province in China (No.JWFX2024010).

## Declaration of competing interest

The authors declare that they have no known competing financial interests or personal relationships that could have appeared to influence the work reported in this paper.
